# Resonant Acoustic Spectroscopy for Measuring Complex Modulus of Bitumen

**DOI:** 10.3390/s26020720

**Published:** 2026-01-21

**Authors:** Frederik A. Kollmus, Lucas Sassaki Vieira da Silva, Michael P. Wistuba

**Affiliations:** Braunschweig Pavement Engineering Centre, Technische Universität Braunschweig, 38106 Braunschweig, Germany; lucas.silva@tu-braunschweig.de (L.S.V.d.S.); m.wistuba@tu-braunschweig.de (M.P.W.)

**Keywords:** Resonant Acoustic Spectroscopy (RAS), complex modulus, asphalt binder rheology, Dynamic Shear Rheometer

## Abstract

**Highlights:**

**What are the main findings?**

- Resonant Acoustic Spectroscopy (RAS) was successfully applied to bitumen for the first time and can determine the complex modulus of bitumen in a temperature range from −30 °C to 20 °C.
- At low temperatures, RAS-derived complex modulus values show good agreement with Dynamic Shear Rheometer (DSR) measurements, especially at −20 °C and −30 °C.

**What are the implications of the main findings?**

- RAS provides a fast, cost-effective, and non-destructive method for assessing stiffness and ageing effects in bitumen, offering potential for quality control and routine material evaluation.
- Due to the determination and use of natural resonant frequencies, RAS alone cannot generate a full master curve of bitumen and is not suitable for characterizing bitumen behaviour at high temperatures or low loading frequencies.

**Abstract:**

The complex modulus is one of the intrinsic properties of bituminous materials, and, hence, is of importance for their rheological characterization. It was shown by various authors that the complex modulus of asphalt mixtures can be calculated from dynamic modulus measurements using the Resonant Acoustic Spectroscopy (RAS). This paper extends the RAS technique to bitumen. For the purpose of validation, rheological data for the same bitumen are also derived from standard Dynamic Shear Rheometer (DSR) tests, and the master curves resulting from both methods are compared. The laboratory programme comprised a temperature range from −30 °C to 20 °C, and four different bitumens in unaged and aged condition, resulting in 36 different test variants. RAS successfully characterizes the complex modulus of bitumen and reflects temperature and ageing effects, with good agreement to DSR results at low temperatures. At higher temperatures, viscosity and damping introduce deviations, indicating that RAS is effective for modulus evaluation but not sufficient for complete master curve development.

## 1. Introduction

The complex modulus is a key material property in material science, also used to characterize viscous materials, such as asphalt mixtures, bitumens, and other viscous binders. The complex modulus is the contribution of the material to the stiffness of the specimen tested (its stiffness is also determined by the geometry of the loading layout) and can be determined through dynamic excitation of the material in normal loading mode (usually referred to as E*) but also in shear loading mode (usually referred to as G*). Dynamic loading can be generated either in *cyclic mode*, like in a Dynamic Shear Rheometer (DSR), or in *vibratory mode*, like in Resonant Acoustic Spectroscopy (RAS), with much higher frequencies than in a DSR.

During the test, stress and strain signals are recorded in functions of temperature and time (frequency). The time shift between the stress and the strain phases is referred to as the phase angle, which is key to the viscous response of the material. These data form the complex modulus in the functions of temperature and frequency, i.e., stress function, strain function, and phase angle.

If the load amplitude is small and the number of load repetitions is low, asphalt mixtures and bitumens show a linear viscoelastic (LVE) material behaviour with good approximation [1]. Within the LVE range, the material parameters of the complex modulus can be determined independently of the load magnitude and loading type [2,3]. The absolute value of the complex modulus can be simply represented by the ratio of the stress and the strain amplitudes (see, e.g., [1]).

Today, the measuring device usually adopted for determining the complex modulus of bitumens is the Dynamic Shear Rheometer (DSR). By means of the DSR, the complex shear modulus and the phase angle of bitumens can be accurately determined in *cyclic* oscillatory shear mode in a frequency range of 0.01 to 30 Hz, and in a temperature range from approximately −30 to 90 °C [4,5].

The *vibratory test* method, used in this study, is called Resonant Acoustic Spectroscopy (RAS), also known as the Impact Resonance Test (IRT), Free-Free Column Test, or Acoustic Resonance Test.

RAS is based on wave propagation within the material, and is fast and easy to perform, economical, and definitely non-destructive [6]. “The principle of this method is based on the fact that a part’s physical structure causes it to have a unique frequency of oscillation. Every part with the same physical properties will have the same vibration properties, and any flaw will cause this vibration ‘fingerprint’ to change” [7]. Waves are produced by either hitting the specimen externally using an impact hammer [8,9] or by applying sound waves [10,11,12].

RAS is widely used for various materials, such as steel, concrete, or glass. Also, it has been successfully used to detect metal damage in the automotive industry for many years [13]. It is also an established method to characterize cement concrete materials, for determining the dynamic modulus but also for detecting cracks in structures [14]. Testing principles for cement concrete materials are standardized within the American standard ASTM C215 [15], including recommendations for specimen dimensions.

More recently, the application of RAS to asphalt mixtures was introduced. Various authors reported on the successful determination of the complex modulus of asphalt mixtures at a range of temperatures. Good correlation with results from conventional test methods were reported [9,16,17,18,19,20,21,22,23,24,25,26,27,28].

Based on suitable dimensions of cement concrete specimens, various geometries of asphalt specimens were analyzed through RAS, including beam geometries [9,20,24] and cylindrical specimens cored from asphalt slabs or compacted by gyratory compactor produced in a laboratory [19,21,25,29]. A working group has been recently set up within the International Union of Laboratories and Experts in Construction Materials, Systems and Structures (RILEM) to further develop the application of RAS to construction materials for road construction [30]. However, RAS is still not a standard test method for asphalt materials.

The objective of this study is to assess the application of RAS to bitumen. For this purpose, four usual bitumens (two plain bitumens and two polymer-modified bitumens) are tested at three different ageing stages:

- Fresh/unaged;
- Short-term aged using the rolling thin-film oven test (RTFOT-aged) according to EN 12607-1 [31];
- Long-term aged using the pressure ageing vessel in addition to RTOFT (RTFOT+PAV aged), according to EN 14769 [32].

**Figure 1:**
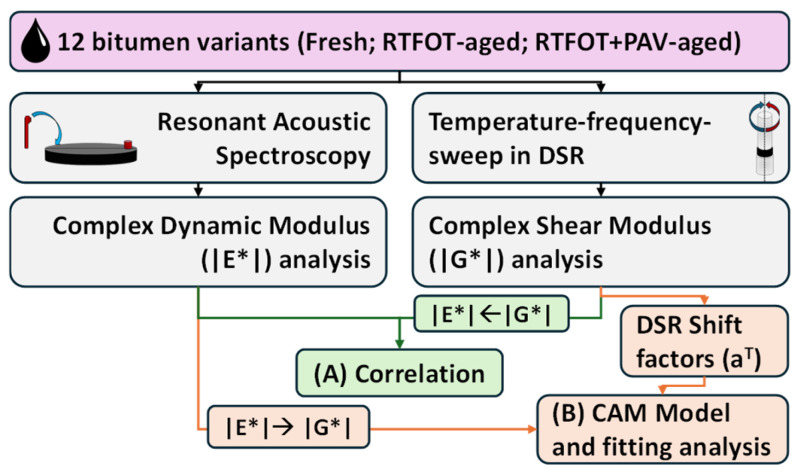
Flow-chart of the experimental programme of this study. RTFOT…Ageing through rolling thin-film oven test (RTFOT; according to EN 12607-1 at 163 °C for 75 min); RTFOT+PAV…Additional ageing in pressure ageing vessel (PAV; according to EN 14769); (A)…Checking the correlation between measurements with Resonant Acoustic Spectroscopy (RAS) and Dynamic Shear Rheometer (DSR); (B)…Using the Christensen–Anderson–Marasteanu model (CAM model) for Master curve construction.

A total of three specimens of each ageing stage is tested at six temperatures using RAS, resulting in a total number of 216 individual tests (4 bitumens × 3 specimens × 3 ageing × 6 temperatures). The complex modulus resulting from RAS in a temperature range of −30 to 20 °C is always compared to the corresponding result from Temperature–frequency sweep (T-f-sweep) tests using the Dynamic Shear Rheometer (DSR). For this purpose, the master curves are used, as they are obtained from both methods. A flow-chart of the experimental programme of this study is presented in Figure 1.

## 2. Research Background

### 2.1. Relationship Between |E*| and |G*|

**Figure 2:**
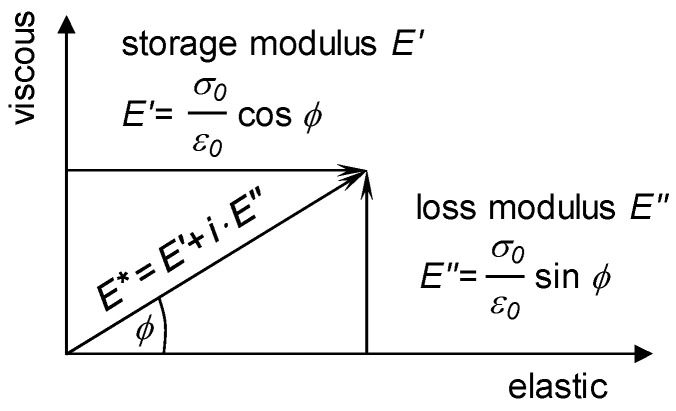
Decomposition of the complex modulus: storage modulus *E′* and loss modulus *E″* [1].

In the Gaussian plane, the complex modulus can be composed of two characteristic parts: the *storage modulus* plotted on the real axis and referred to as *E′* (for normal loading mode, but the same applies to shear mode, see below), which is related to the elastic part of the material behaviour, and the *loss modulus* plotted on the imaginary axis and referred to as *E″*, which is related to the viscous behaviour of the material (see Figure 2). The relationship between the contributions of *E′* and *E″* is determined by the phase angle, which is the time delay between the applied load and the answer of the material in terms of strain in stress-controlled loading mode, or stress in strain-controlled loading mode. It is evident that phase angle values which are higher in comparison to their lower counterparts are indicative of viscous materials; conversely, lower phase angle values are indicative of elastic materials.

The norm of the complex modulus (absolute value) is the length of the vector in the Gaussian plane, denoted as |E*|. This value is generally understood to be the characteristic material stiffness for any bitumen, asphalt mastic, or asphalt mixture, measured in cyclic mode.

The determination of |E*| is described in the American standard AASHTO T342 [33] and the European standard EN 12697-26 [34].

In analogy to the norm of the complex modulus |E*|, the norm of the complex shear modulus |G*| can be determined in shear mode using the Dynamic Shear Rheometer (DSR) [35], where a tangential rotary-oscillating shear load acts on a cylindrical specimen.

In the LVE domain, both parameters are directly linked and can be calculated one from another, whereby they always depend on the temperature and frequency. Strictly speaking, this relationship is defined for the complex moduli E* and G* under LVE conditions. In this study, Equation (1) is applied to the absolute values |E*| and |G*| as an approximation, since the analysis focusses on the determination of the stiffness parameters. The relation between |E*| and |G*| in the LVE domain is given by Equation (1), governed by the Poisson ratio (ν).

(1) $$|G^{*}|=\frac{\left|E^{*}\right|}{2\cdot (1+\nu )}$$

The Poisson ratio *ν* is not a constant value (0.5 is often stated in the literature). It varies between about 0.35 for high loading frequencies and/or low temperatures, and 0.5 for low loading frequencies and/or high temperatures [36]. Furthermore, Kim et al. [37] determined ν using the Tension–Torsion test performed directly at the same specimen using the DSR. In this study, the Poisson ratio was considered as a function of temperature when calculating the storage modulus. The Poisson ratios given in Table 1 apply to bitumen 50/70 at different temperatures. For the sake of simplicity, these were also used for the bitumens in this study.

### 2.2. Background of Resonant Acoustic Spectroscopy (RAS)

A resonance frequency occurs in a solid material if the frequency of an acting force is equal to the natural frequency of the material. The natural frequency depends upon the mass, the dimensions, and the elastic constants of the material, if it has free boundary conditions [6,38].

The basic operational principle of a resonance frequency measurement is to induce a vibratory excitation in the specimen and to measure its dynamic response. Excitation is achieved by, for example, manually hitting on the edge of the specimen with a small impact hammer. The induced vibration goes through the specimen because of wave propagation. The wave propagation depends on the stiffness and the density of the material, but also on its temperature [6]. The vibration reaches the opposite side of the specimen, where the acceleration response of the specimen is measured with an accelerometer.

**Figure 3:**
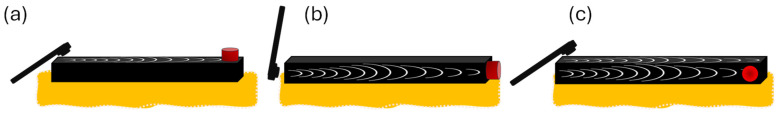
Wave propagation modes: (**a**) flexural, (**b**) longitudinal, and (**c**) torsional.

Three different modes of wave propagation/excitation directions can be applied to a cylindrical or prismatic specimen, namely flexural, longitudinal, and torsional modes, as shown schematically in Figure 3 (see [15] for cement concrete materials).

In this study, the flexural mode is applied to cylindrical specimens of bitumen as it forms the basis for Equation (10).

**Figure 4:**
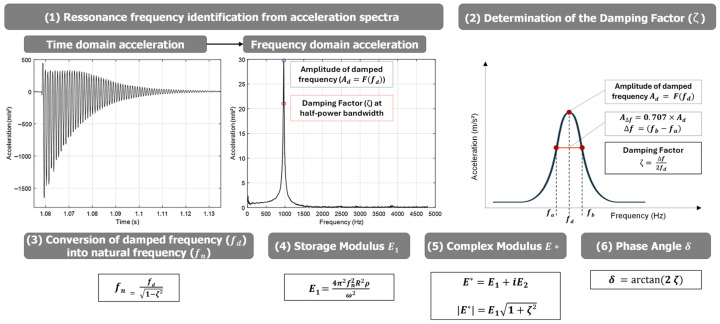
Summary of steps to determine the complex modulus |E*| from RAS divided in steps of (1). Resonance frequency identification, (2) Damping factor determination, (3) Conversion of damped frequency into natural frequency, Determination of (4) Storage Modulus, (5) Complex Modulus, and (6) Phase Angle. Damping factor determination is marked in red to highlight from other information.

The output signal of the accelerometer is recorded in the unit of m/s^2^ (see Figure 4). After measuring the acceleration, the frequency response curve is obtained by transferring the time domain into the frequency domain using Fast-Fourier Transformation (FFT) [39,40].

For a given time-domain signal such as acceleration, the resulting spectra can be written in the form of the Complex Fourier Series (Equation (2)):

(2) $$f\left(x\right)=\sum_{k=-\infty }^{\infty }c_{k}e^{\frac{\mathrm{ik}\pi x}{L}}$$

where $c_{k}=\frac{1}{2L}\int_{-L}^{L}f(x)e^{\frac{-\mathrm{ik}\pi x}{L}}\mathrm{dx}$ and $\frac{k\pi }{L}$ is substituted by $\omega_{k}$; therefore, $∆\omega =\frac{\pi }{L}$.

For the given expression, if L→∞ in the interval of −∞ to +∞, the expression becomes a Riemann Integral, and Equation (3) can be simplified to Equation (4):

(3) $$f\left(x\right)=\int_{-\infty }^{\infty }\frac{1}{2\pi }\int_{-\infty }^{\infty }\frac{1}{2\pi }f(\xi )e^{-i\omega \xi }d\xi e^{i\omega x}d\omega$$

(4) $$f\left(x\right)=\int_{-\infty }^{\infty }\frac{1}{2\pi }\hat{f}\left(\omega \right)e^{i\omega x}d\omega$$

Finally, the Fourier Transformation is denoted by Equation (5):

(5) $$\hat{f}\left(\omega \right)=F(f\left(x)\right)=\int_{-\infty }^{\infty }f\left(x\right)e^{-i\omega x}\mathrm{dx}$$

The result of this process is the amount, amplitude, or magnitude of the acceleration for a given frequency within the analyzed range denoted by ($\hat{f}\left(\omega \right)$). In the subsequent analyses of this study, the result will be referred to as the “FFT Magnitude”. Furthermore, it is possible to obtain the inverse function from the frequency domain to the time domain, which can then be formulated by Equation (6):

(6) $$f\left(x\right)=F^{-1}(\hat{f}\left(\omega )\right)=\frac{1}{2\pi }\int_{-\infty }^{\infty }\hat{f}\left(\omega \right)e^{i\omega x}d\omega$$

### 2.3. Determination of Complex Modulus with Half-Power Bandwidth Method

The half-power bandwidth method applied in this study is based on the classical theory of linear single-degree-of-freedom (SDOF) systems. Its validity requires linear material behaviour, low-to-moderate damping, and dominance of a single resonance mode in the vicinity of the evaluated peak (see Figure 4). All measurements were performed within the linear viscoelastic domain, and the analyzed resonance peaks were sufficiently separated from adjacent modes.

According to Clough and Penzien [41], the measured resonance frequencies are the damped natural frequencies *f_d_* of the specimen. These measured resonance frequencies are converted to natural frequencies *f_n_*, applying the damping ratio *ξ* (Equation (7)):

(7) $$f_{n}=\frac{f_{d}}{\sqrt{1-\xi^{2}}}$$

The damping ratio *ξ* is determined with the half-power bandwidth method (Equation (8)):

(8) $$\xi =\frac{\Delta f}{2f_{d}}$$

where Δ*f* is the width of the frequency response curve at 1/√2 (half power) of the maximum amplitude of the curve. The damping ratio is also used to determine the phase angle *φ* (Equation (9)):

(9) φ=arctan(2·ξ)

“This procedure of estimating damping is applicable as long as the damping ratio does not exceed approximately 0.5 (half of the critical damping) and the recorded length of the signal is longer than the inverse of the bandwidth (Δ*f*)” [42], which corresponds to the low-to-moderate damping conditions typically assumed for the half-power bandwidth method.

According to Martinček [43] the storage modulus *E’* can be obtained from Equation (10):

(10) $$E^{\prime }=\frac{4\pi^{2}f_{n}^{2}R^{2}\rho }{\varpi^{2}}$$

where *R* is the radius of the specimen; *ρ* is the density of the specimen; and *ϖ* is a material parameter.

The material parameter *ϖ* can also be determined by interpolation, according to Martinček [43], for the first natural vibration in the flexural mode, related to the height and the radius of the specimen and Poisson’s ratio *ν*. The relation between the damping ratio and modulus can be expressed by Equation (11) [41]:

(11) $$\xi =\frac{E^{\prime \prime }}{2E^{\prime }}$$

where *E*″ is the loss modulus.

The norm of the complex modulus |E*| can then be determined by Equation (12).

(12) $$\left|E^{*}\right|=|E^{\prime }+iE^{\prime \prime }|=E^{\prime }\sqrt{1+4\xi^{2}}$$

The determination of the complex modulus is summarized in Figure 4, considering all the presented steps from data acquisition to final complex modulus values.

**Figure 5:**
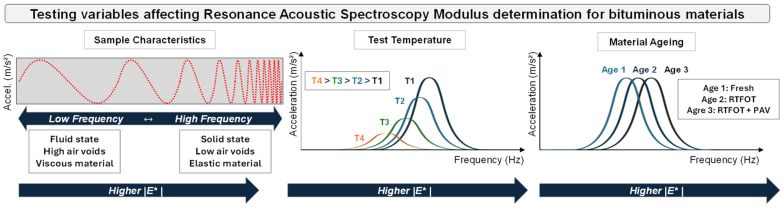
Testing variables affecting RAS divided into sample characteristics, testing temperature, and material ageing and how it modifies the resonant frequency.

The material properties as well as the testing conditions affect the specimen response and modulus resulting from RAS tests [6,27]. This is especially true for viscoelastic materials susceptible to temperature and frequency of excitation, such as bitumen, asphalt mastics (mixture of bitumen and aggregate fines of grain sizes up to 0.063 mm), and asphalt mixtures, which also change their internal properties due to ageing. The relation of the aforementioned variables is illustrated in Figure 5.

## 3. Materials and Testing Protocols

### 3.1. Bitumens

Table 2 provides an overview of the bitumen samples tested in this study in terms of penetration and softening ring and ball values, in fresh and in aged states.

### 3.2. Dynamic Shear Rheometer: T-f-Sweep Tests and Master Curve Modelling

During the Temperature–frequency sweep (T-f-sweeps) tests over a temperature range of −30 to 90 °C, the temperature is increased in increments of 10 °C. The conditioning time for every temperature was defined based on previous experience in the research group. The test frequency of the oscillatory stress varies between 0.1 and 10 Hz. Suitable loading is selected based on previously performed amplitude sweeps [35] for each temperature, ensuring that the test remains within the LVE range, and, therefore, is non-destructive testing [46]. For this purpose, the following DSR geometries are used:

- The 4 mm plate–plate geometry (gap 2.00 ± 0.01 mm) in a temperature range of 30 °C to 0 °C;
- The 8 mm plate–plate geometry (gap 2.00 ± 0.01 mm) in the range of 10 °C to 40 °C;
- The 25 mm plate–plate geometry (gap 1.00 ± 0.01 mm) in the range of 30 °C to 90 °C.

According to [47], when using the 4 mm plate–plate geometry, the gap is corrected during thermal stabilization and kept constant during the test.

The resulting rheological parameters (complex shear modulus G* composed of storage modulus *G′*, loss modulus *G″*, and the corresponding phase angle) are then used to generate master curves. The master curve is created using the Time–Temperature Superposition Principle (TTSP, ref. [48]) with the shift factors obtained with the WLF function (Equation (13)) [49], which translates the modulus at a given temperature into the reference temperature.

(13) $$\log\left(\alpha_{T},T_{\mathrm{ref}}\right)=-\frac{C_{1}(T-T_{\mathrm{ref}})}{C_{2}+(T-T_{\mathrm{ref}})}$$

where *α_T_* is the temperature shift factor, *T_ref_* is the reference temperature where the other data are translated into the master curve, *T* is the temperature to be translated, and *C*_1_ and *C*_2_ are adjusting constants.

The data for |G*| (see Equation (14)) and *δ* (see Equation (15)) are then modelled using the Christensen–Anderson–Marasteanu model (CAM model, see [50]) (see [51,52,53]):

(14) $$|G^{*}|={|G}_{0}^{*}|+\frac{\left|G_{\infty }^{*}|-|G_{0}^{*}\right|}{{\left[1+{\left(\frac{f_{c}}{f^{\prime }}\right)}^{k}\right]}^{\frac{m_{e}}{k}}}$$

where |G*| is the norm of the complex shear modulus, $\left|G_{0}^{*}\right|$ is when the frequency tends to zero, $\left|G_{\infty }^{*}\right|$ is when the frequency tends to infinity, $f_{c}$ is a location parameter with dimensions of frequency, $f^{\prime }$ is the reduced frequency, *k* and *m_e_* are shape parameters, and *δ* is the phase angle (Equation (15)):

(15) $$\delta =90l-(90l-\delta_{m}){\left\{1+{\left[\frac{\log(\frac{f_{d}}{f^{\prime }})}{R_{d}}\right]}^{2}\right\}}^{-\frac{m_{d}}{2}}$$

where $\delta_{m}$ is the phase angle at $f_{d}$, *R_d_* and *m_d_* are shape parameters, and *l* is a variable parameter which equals 0 when $f<f_{d}$ and 1 if $f\geq f_{d}$.

### 3.3. RAS: Specimen Preparation

**Figure 6:**
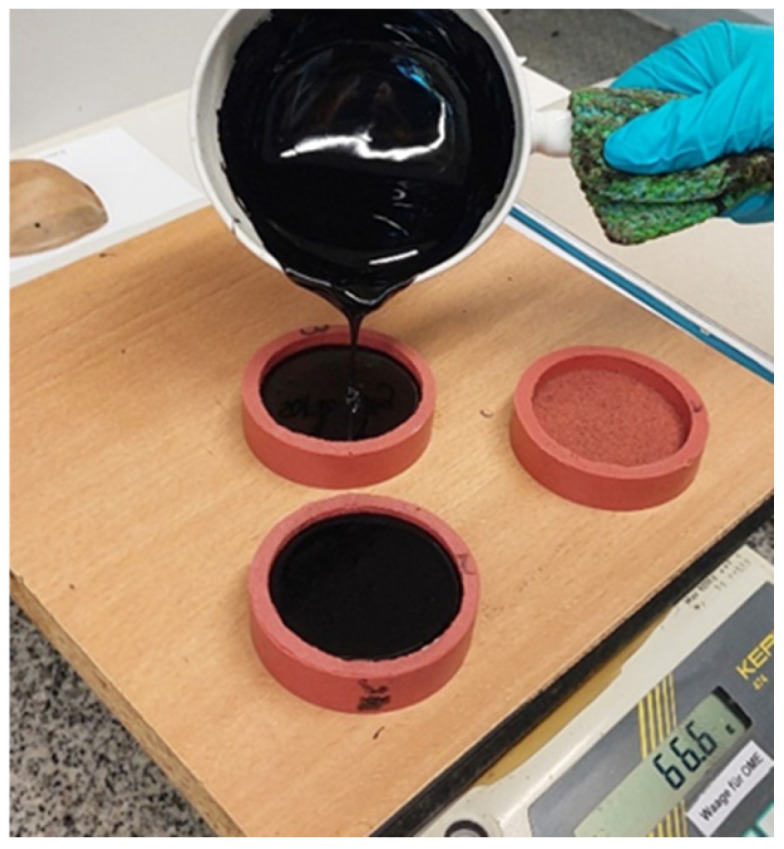
RAS specimen preparation.

Sampling of the bitumens for RAS testing is in accordance with European standard EN 58 [54], and specimens are prepared in accordance with EN 12594 [55]: the heated specimen is homogenized by stirring with a glass rod, and then 30 g is filled without bubbles into a mould made of silicone (see Figure 6). The specimen is then cooled to ambient temperature and kept covered for a minimum duration of 2 h. Afterwards, the specimen is cooled to a temperature of −18 °C for a duration of 30 min, so that it can easily be demoulded from the silicone mould (see [47] Büchner et al., 2021).

### 3.4. RAS: Test Set-Up and Procedure

**Figure 7:**
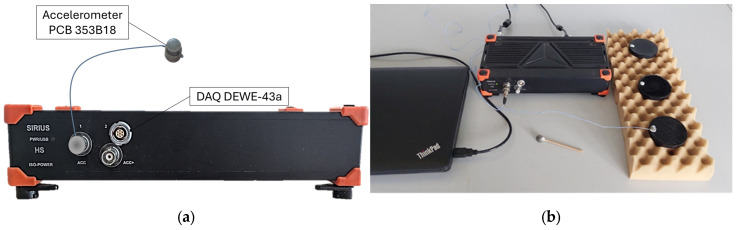
(**a**) Data Acquisition Device DEWE-43a with accelerometer PCB 353B18; (**b**) test assembly with the specimens on the soft foam bed.

The RAS test set-up used in this study includes a self-made impact hammer, with an impact mass of 4.7 g, to apply the load manually. An accelerometer of the type PCB model 353B18 is used to measure the vibrations over time. The weight of the accelerometer is 1.8 g, so that the response of the specimen is not affected [56]. The sensitivity of the accelerometer sensor is 1.02 mV/(m/s^2^), according to the producer. The accelerometer is directly connected to a Data Acquisition Device (DAQ). The DAQ used in this study is of the type DEWE-43a (see Figure 7a).

The impact hammer is not connected to the DAQ, because the force of the impact has no effect; according to Gudmarsson [57] the “resonance frequencies nor the amplitude seem to depend significantly on the magnitude of the applied load”.

The DAQ is set to a constant sampling rate of 200 kHz and converts the analogue signals to digital signals. To conduct the measurements and record the data, a data acquisition toolbox of the software type DEWEsoft X3 is used. The toolbox is compatible with the DAQ and enables us to perform FFT analysis in real time. According to Gudmarsson [9], the specimen is best placed on a soft foam for free boundary conditions, which is an essential requirement for this method (see Figure 7b).

RAS is performed for each specimen (36 test variants in total) at six temperatures, starting at a temperature of −30 °C and increasing in steps of 10 °C to the final temperature of 20 °C. At each test temperature, the time for temperature conditioning is always kept for at least 90 min. The measurements are performed with specimens located inside a temperature chamber. However, the door of the chamber is opened for a duration of approximately 5 to 10 s to manually apply the load. The opening may cause a small change in air temperature inside the chamber, while the temperature change in the specimen is neglected.

Each specimen is hit with the impact hammer at least five times within approximately 5 to 10 s, the acceleration over time is recorded, and the average result of the five hits is used for modulus determination.

As mentioned before, FFT analysis can be directly processed in real time by using the toolbox or afterwards using Matlab.

## 4. Results and Evaluation

### 4.1. Rheological Characterization of Bitumens

**Figure 8:**
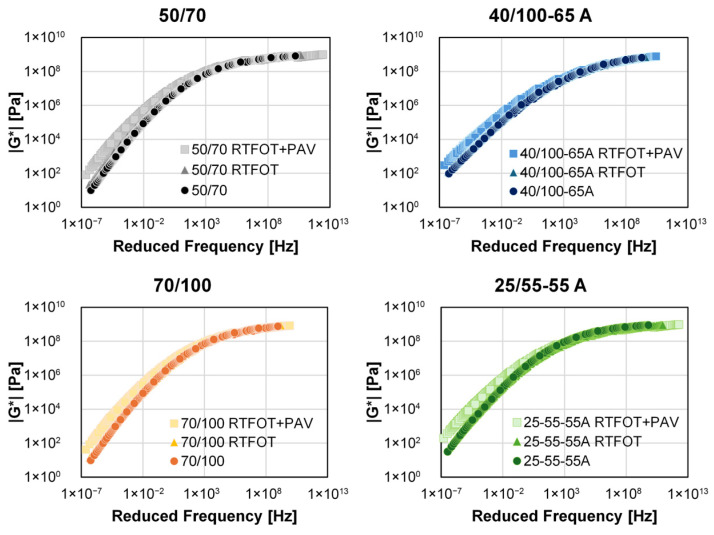
Master curves for all bitumen variants at the reference temperature of 20 °C determined using the T-f-sweep test in the DSR and modelled by the CAM model and WLF parameters.

As illustrated in Figure 8, the master curves of all bitumen variants are based on the data from T-f-sweep tests translated to a reference temperature of 20 °C. The master curves are fitted with the CAM model, and are necessary for further comparison with RAS results.

As expected, aged specimens present a significant increase in |G*| values for the entire observed spectra. This observation is also represented in Table 3 by the CAM model’s parameters and WLF parameters for the four bitumens in fresh and aged conditions.

Particularly noticeable are the changes in the modelling variables in the WLF equation, which reflect the sensitivity of the material to temperature changes. In the CAM model, which describes the shape of the master curve, see, in particular, the parameter *fc*, which is a frequency-dependent variable decreasing for the aged conditions of every neat bitumen type and increasing at the RTFOT condition, followed by decrease for the polymer-modified bitumens.

### 4.2. Moduli Obtained from RAS

**Figure 9:**
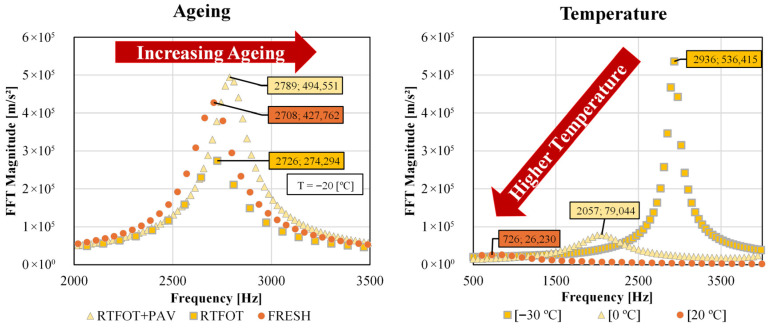
Effects of ageing and temperature on the FFT acceleration magnitude of bitumen.

When testing with RAS, it should be noted that the material properties can change the acceleration amplitude and the value of the first natural frequency. Figure 9 (left) shows the results for the same bitumen at the same temperature (−20 °C) for the fresh, RTFOT-aged and RTFOT+PAV-aged conditions. Figure 9 (right) shows the results for a fresh bitumen, but at different temperatures (−30 °C, 0 °C, and 20 °C).

It can be seen that the value for the first resonant frequency of the material is also dependent on the ageing of the material (see Figure 9, left), among other things, like the temperature or the geometry of the specimen. The more aged the material is, the higher is the first resonant frequency, due to the reduction in damping as a result of an increase in the elastic components of the bitumen. This result was to be expected, as similar properties had previously been observed in samples of asphalt mixtures [58].

Furthermore, Figure 9 (right) shows that an increase in temperature leads to a significant reduction in the first resonant frequency, as the increase in viscous components in the bitumen leads to increased damping. This increased damping can also be seen in the increasing compression of the curve of the first resonant frequency with increasing test temperature.

However, the presented study underlines the effectiveness of the method in detecting material changes caused by chemical and physical modifications of the bitumen specimens.

**Figure 10:**
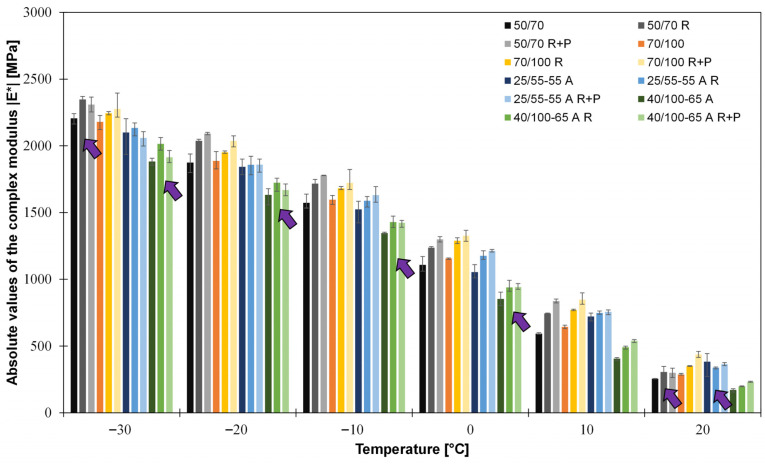
Complex modulus results obtained from RAS at different temperatures (−30 °C to 20 °C) with purple arrows added where the value for |E*| does not increase with increasing age to highlight some of the exceptions to the expectation.

Figure 10 shows the norm (absolute value) of the complex modulus |E*| of all investigated bitumens at all test temperatures, as obtained from RAS. The absolute deviations from the mean values are also displayed.

Generally, it can be stated that the absolute value |E*| decreases with increasing temperature. With increasing ageing level, the value increases. This is true for almost all four bitumens in all temperatures; an exception is observed for specimens indicated with a purple arrow in Figure 10. At −30 °C it can be seen that there is no clear trend towards an increase in the values for |E*| due to ageing, and one of the reasons is due to the low-temperature asymptotic behaviour of the |E*| of bitumen, which can be seen clearer in the master curve modelling.

**Figure 11:**
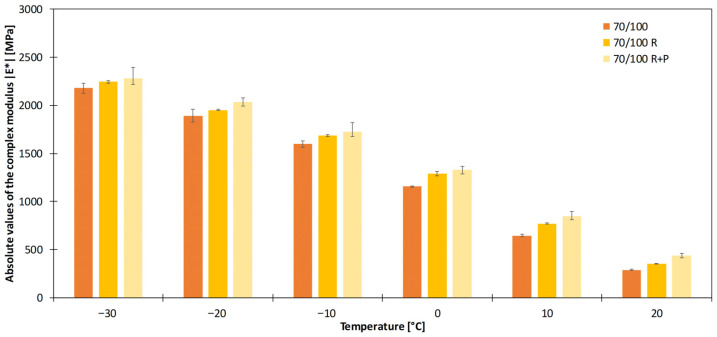
Absolute value of complex modulus for 70/100 plain bitumen, and fresh and aged variants (R…RTFOT, R+P…RTFOT+PAV).

As an example, Figure 11 shows the increase in |E*| for the two ageing levels used in this study (RTFOT and RTFOT+PAV).

### 4.3. Correlating RAS with DSR Results

**Figure 12:**
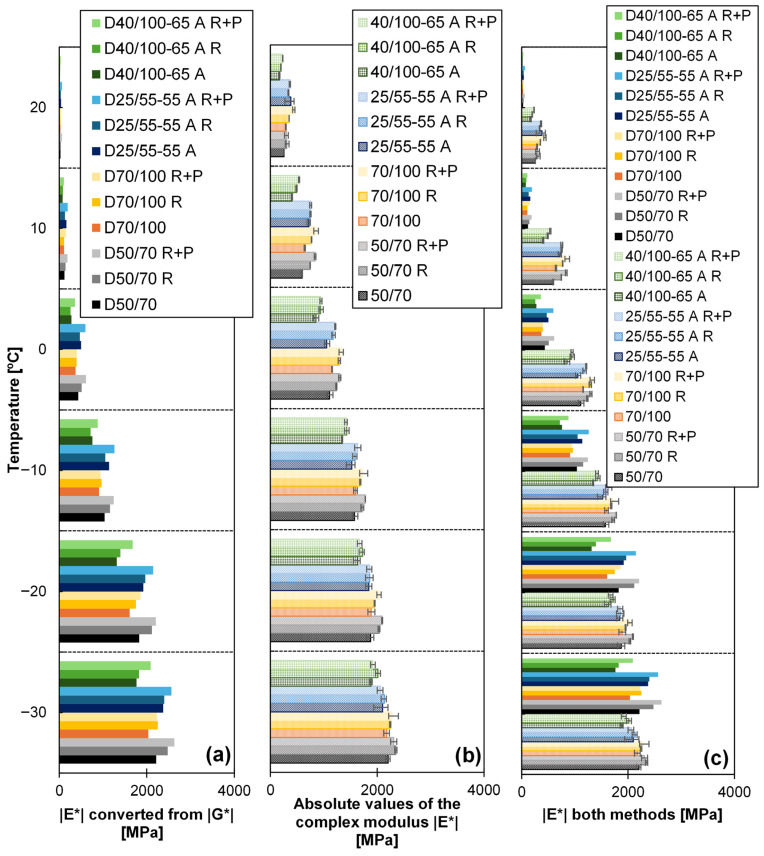
Results of (**a**) |E*| converted from |G*| obtained by T-f-sweep in the DSR, (**b**) |E*| obtained from RAS, and (**c**) both methods.

Figure 12a compares the values for |E*| determined by the following:

- RAS.
- T-f-sweep tests in the DSR at a frequency of 10 Hz. Equation (1) and Poisson ratios from Table 1 were applied to convert |G*| into |E*|. They are labelled with “D” at the beginning.

The values are largely close to each other, but the deviation depends on the temperature.

The results obtained by RAS also show that the method is suitable for differentiating between the various materials tested (see Figure 12b). As also demonstrated in Figure 12b, the RAS results indicate that, at both temperatures (20 °C and 10 °C), the moduli bars exceed those observed in Figure 12a.

A direct comparison is presented in Figure 12c. It shows that at the temperatures of −30 °C and −20 °C, the complex modulus |E*|, as ascertained by RAS, and the converted modulus |E*| are approximately equivalent for tests conducted at temperatures lower than −10 °C.

**Figure 13:**
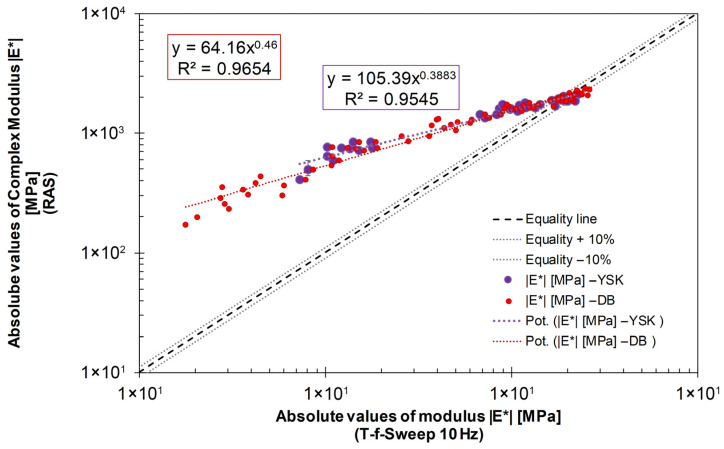
Correlation of |E*| from RAS and T-f-sweep test results at 10 Hz from DSR (YSK… with data for Poisson Ratio from Kim et al. (2024) [37] (see Table 1); DB… with data for Poisson Ratio from Di Benedetto et al. (2007) [36] (see Table 1)).

In order to show this correlation in the low-temperature range and the increasing deviations with increasing test temperature, the converted moduli of all bitumens from the DSR T-f-sweep tests at 10 Hz and the moduli determined by using RAS are compared in Figure 13. This comparison is performed at 10 Hz, as this is the highest tested frequency in the DSR and, accordingly, the higher values for |G*| can be determined.

It should be noted that the complex moduli derived from the first resonance frequency in the RAS measurements correspond to an effective excitation frequency that varies with temperature, material state, and specimen properties.

The data from RAS and from DSR are correlated by a linear function with a coefficient of determination of at least 0.95 (see Figure 13). On the one hand, the values for |E*| (purple boxes in Figure 13) are given using the Poisson ratio according to [36] and, on the other hand, the values for |E*| (red dots in Figure 13) are given using the Poisson ratio according to [37]. With both approaches, a linear relationship between the data determined using RAS and DSR can be seen.

However, the values obtained from RAS are approximately 1000 MPa higher than the DSR-converted values. This can be clearly seen in the figure by the deviation of the trend line from the “equality line”. The values are almost identical at temperatures of −20 °C and −30 °C, but increasingly diverge at temperatures above 0 °C.

These deviations observed in the higher temperature range can be attributed to the increasing frequency dependence of the complex modulus of bitumen. However, Martinček’s [43] model for determining the complex modulus using RAS is based on the principles of elastic material behaviour. Consequently, it can be assumed that the model becomes invalid at higher temperatures due to the higher viscosity of bitumen. In addition, natural frequencies in a range of more than 1000 Hz are measured when performing RAS.

During the T-f sweep test in the DSR, the bitumen in this study is loaded in a significantly lower frequency range of 0.1 Hz to 10 Hz. This complicates the direct comparison of the moduli resulting from RAS and DSR, particularly at elevated temperatures.

Consequently, the comparison between RAS- and DSR-derived moduli does not represent a direct frequency-by-frequency equivalence, but rather a comparison of corresponding stiffness levels and trends. At low temperatures, where the frequency dependence of bitumen is reduced and the material response approaches elastic behaviour, a close agreement between both methods is observed, whereas increasing deviations occur at higher temperatures.

### 4.4. Master Curve Construction with RAS and DSR Data

Due to the fundamentally different excitation principles and frequency domains of RAS and DSR, the construction and comparison of master curves require particular attention to the effective frequency range represented by each method.

**Figure 14:**
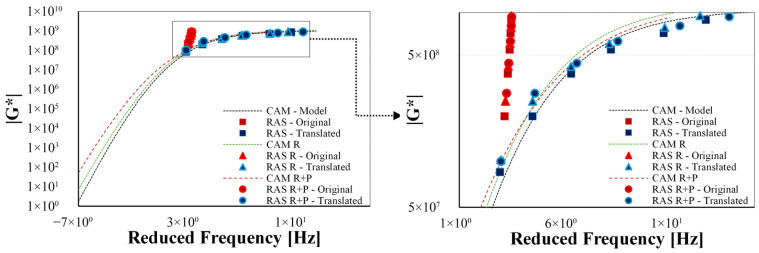
CAM model master curves of 50/70 bitumen with data from DSR and RAS converted |G*| values shift representation where the dashed lines in black for the fresh bitumen, green for RTFOT aged bitumen, and red dashed for RTFOT+PAV aged bitumen while the icons in red are for the data in the calculated frequency while icons in blue are for the data in the translated frequency, the squares are used for fresh bitumen, triangles for RTFOT aged and circles for RTFOT+PAV aged bitumens.

To overcome the difficulty of a direct comparison between the methods used, two master curves are plotted. On the one hand, the master curve of |G*| obtained from data determined by DSR and, therefore, the CAM model regression, is presented in Figure 14. On the other hand, the master curve obtained from the data determined by RAS is presented (also see Figure 14). Both are generated at a reference temperature of 20 °C for the 50/70 bitumen at all three different ageing stages.

To obtain the RAS master curve, the data determined using RAS in a temperature range from −30 °C to 20 °C are converted into |G*| using the *ν* values according to [36] from Table 1. The shifting of the converted RAS data is carried out using the WLF shift factors from Table 3.

**Figure 15:**
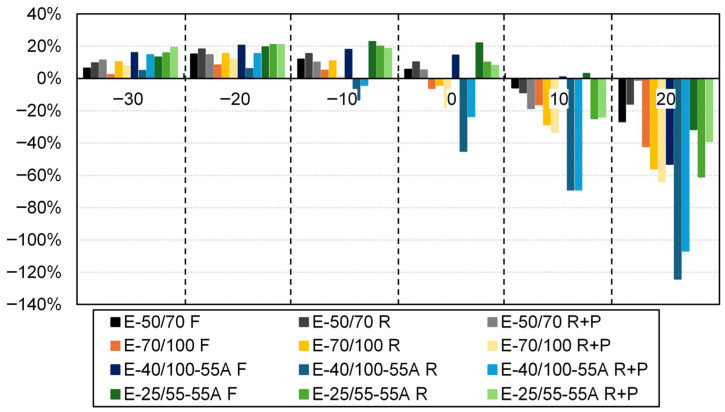
Differences between CAM model fitting from DSR, and RAS converted points for all variants at the tested temperatures from -30 °C to 20 °C.

It can be observed that, despite the implementation of DSR shift factors, a disparity remains between the translated points and the curves. A more effective comparison can be made by referring to Figure 15, in which the RAS moduli are juxtaposed with the modelled values from the DSR master curves at the same frequency. As previously demonstrated, a (passable) margin of error is observed for all evaluated bitumens and ageing conditions up to −10 °C.

Again, the master curve determined with data from RAS, which are converted into complex shear moduli, agrees well with the master curve determined with data from the DSR (see Figure 15). Due to the high natural frequencies determined with RAS, there are more data points at higher reduced frequencies.

It should be noted that it is not possible to determine values for low frequencies and high temperatures with RAS, as it is not possible to test in a temperature range of more than 20 °C. Consequently, it is not possible to derive a master curve from RAS measurements alone. It is obvious that the high temperature/low frequency range is missing fordetailed modelling. Furthermore, only a single frequency (the natural frequency) can be used for each temperature. This makes it difficult to apply the principle of time-temperature superposition using RAS data only.

## 5. Summary and Conclusions

In this study, four different bitumens (50/70, 70/100, 25/55-55 A, and 40/100-65 A) were tested in fresh condition and after short-term ageing in the laboratory using the rolling thin-film oven test (RTFOT) and after additional long-term ageing (RTFOT + pressure ageing in a vessel (PAV)) in the laboratory.

The complex moduli of the bitumens were investigated through two different testing techniques. On the one hand, Resonant Acoustic Spectroscopy (RAS) was used in a temperature range from −30 °C to 20 °C; on the other hand, the Dynamic Shear Rheometer (DSR) was used to run Temperature–frequency sweep (T-f-sweep) tests in a temperature range from −30 °C to 90 °C.

Subsequently, a comparison was made between the data obtained by RAS at the natural vibration frequencies, and obtained by the T-f-sweep DSR test at a frequency of 10 Hz. The correlation of the respective data was then investigated. Furthermore, master curves were modelled using the CAM model and the WLF formulation for data obtained from both test methods.

From the results, the following conclusions are drawn:

- For the first time, RAS was successfully applied for the purpose of bitumen characterization. The method can be used to determine the complex modulus of bitumen at temperatures below 20 °C.
- The absolute value of the complex modulus of the investigated bitumens obtained by RAS is comparable to the value obtained by the DSR (T-f-sweep tests at 10 Hz) at temperatures lower than 0 °C.
- Due to the temperature-dependent viscous nature of bitumen, the absolute value of the complex modulus decreases with increasing temperature. Furthermore, the absolute values of the complex modulus increase in consequence of laboratory ageing. Both properties can be clearly detected also by means of RAS.
- The complex moduli obtained from RAS correlate with the DSR data from T-f-sweep tests, particularly at low temperatures, where the frequency dependence of bitumen is reduced and the material response approaches elastic behaviour. In general, larger absolute values of complex modulus are obtained with RAS, especially at temperatures higher than −10 °C. The lower the test temperature, the higher is the convergence among the two methods, which are more or less identical at a temperature of −30 °C. This observation is mainly related to the decreasing dependence of the bitumen on the test frequency at lower temperatures as it approximates to an elastic material.
- During the T-f-sweep test in the DSR, the bitumen is loaded in a frequency range of 0.1 to 10 Hz. However, by means of RAS (natural) resonant frequencies higher than 1000 Hz are measured. Accordingly, the low frequency part of the bitumen master curve cannot be designed using only data determined by means of RAS because of the inherent limitation to use the natural resonant frequencies of the specimen. The measured frequencies can be somewhat reduced by using larger samples, but will still be higher compared to the frequency range used in the T-f-sweep test in the DSR.
- It is not possible to determine the master curve only from RAS, because values are missing at higher temperatures and more frequencies are needed. By using the bitumen shift factors (WLF factors) from DSR tests, it is possible to shift RAS data into a master curve for the intermediate and high-frequency portions (temperatures from −30 °C to 20 °C).
- While RAS performs well in the elastic or low-temperature area, its limitations in the viscous or high-temperature range suggest that future studies should explore hybrid approaches combining RAS results with a limited set of DSR data to construct master curves more efficiently than using DSR measurements alone.
- A limitation of the present study is the use of a constant Poisson’s ratio derived from bitumen 50/70 for all tested variants; measuring Poisson’s ratios for polymer-modified and aged bitumen is recommended in future research to enhance modulus calculation accuracy.

In conclusion, the presented study shows that it is basically possible to use RAS for measuring the complex modulus of bitumens in a temperature range from −30 °C up to 20 °C, but it is not possible to compare it directly with the results from a standard test method by using the DSR. The method for converting the data into each other must be investigated further, especially using other values for the Poisson ratio, along with the master curve construction method using both RAS and DSR data and other ageing methods, such as bitumen extracted from pavement core samples.

Despite the limitations presented here, RAS itself is still a promising technique and further research is needed not only in the asphalt scale but also in the bitumen, mastics, and mortar levels to better comprehend how to address properties using this method. The utilization of RAS for pure bitumen is, in essence, an attempt to obtain a pure material response, given that bitumen is a complex material. The primary application scenario for this technique is evident in the quality control of asphalt mixtures following compaction or a cost-effective approach to moduli determination as design criteria both for bitumen as for asphalt mixtures. This is due to the fact that most of the equipment necessary for this kind of assessment (DSR) is significantly more expensive than the simple system required for RAS testing (thermal chamber, DAQ, and sensor).

## Figures and Tables

**Table 1:** Poisson ratios for different testing temperatures of bitumen 50/70.

Temperature [°C]	Di Benedetto et al. (2007) [36]	Kim et al. (2024) [37]
	Poisson Ratio (ν)	
−30	0.35	-
−20	0.40	0.43
−10	0.45	0.37
0	0.48	-
10	0.50	0.40
20	0.50	-

**Table 2:** Bitumen variants classified by needle penetration (EN 1426 [44]) and softening point ring and ball (EN 1427 [45]).

Bitumen	Age	Property	
		Penetration [10^−1^ mm]	Softening Point Ring and Ball [°C]
50/70	Fresh	57	51.7
	RTFOT ^1^	35	58.0
	RTFOT+PAV ^2^	25	67.5
70/100	Fresh	82	47.8
	RTFOT ^1^	51	53.7
	RTFOT+PAV ^2^	33	63.6
25/55-55 A	Fresh	43	59.1
	RTFOT ^1^	32	64.2
	RTFOT+PAV ^2^	21	74.2
40/100-65 A	Fresh	58	73.2
	RTFOT ^1^	52	73.2
	RTFOT+PAV ^2^	36	78.2

^1^ Ageing through rolling thin-film oven test (RTFOT; according to EN 12607-1 at 163 °C for 75 min); ^2^ Additional ageing in pressure ageing vessel (PAV; according to EN 14769).

**Table 3:** WLF and CAM model parameters for all bitumen variants tested in DSR with T-f-sweep.

Material	WLF			CAM Model					
	C1	C2	T_ref_ (°C)	|G*_e_|	|G*_g_|	f_c_	k	m_e_	R^2^
50/70	15.80	132.69	20.00	0.00	$1.0\times 10^{9}$	5.487	0.155	1.111	1.000
50/70 R	16.19	136.47	20.00	0.00	$1.1\times 10^{9}$	11.689	0.160	1.000	1.000
50/70 R+P	15.69	123.79	20.00	0.00	$1.1\times 10^{9}$	1.014	0.137	1.000	1.000
70/100	17.77	157.15	20.00	0.00	$1.0\times 10^{9}$	7.085	0.156	1.096	1.000
70/100 R	17.38	152.19	20.00	0.00	$1.1\times 10^{9}$	22.390	0.161	1.000	1.000
70/100 R+P	17.41	144.15	20.00	0.00	$1.1\times 10^{9}$	2.400	0.140	1.000	1.000
25/55-55 A	17.75	163.68	20.00	0.00	$1.0\times 10^{9}$	4584.896	0.191	0.714	0.999
25/55-55 A R	15.44	122.44	20.00	0.00	$1.1\times 10^{9}$	1.120	0.109	1.000	0.998
25/55-55 A R+P	15.60	113.55	20.00	0.00	$1.1\times 10^{9}$	0.100	0.100	1.000	0.998
40/100-65 A	17.11	141.66	20.00	0.00	$1.0\times 10^{9}$	21.879	0.168	0.938	1.000
40/100-65 A R	15.33	122.53	20.00	0.00	$1.1\times 10^{9}$	3.807	0.137	1.000	1.000
40/100-65 A R+P	16.24	118.54	20.00	0.00	$1.1\times 10^{9}$	0.203	0.120	1.000	0.999

R … Ageing through rolling thin-film oven test (RTFOT according to EN 12607-1 at 163 °C for 75 min); R+P … additional ageing in pressure ageing vessel (PAV according to EN 14769).

## Data Availability

The original contributions presented in this study are included in the article. Further inquiries can be directed to the corresponding author.
